# Comparison of short and long forms of the Flinders program of chronic disease SELF-management for participants starting SGLT-2 inhibitors for congestive heart failure (SELFMAN-HF): protocol for a prospective, observational study

**DOI:** 10.3389/fmed.2023.1059735

**Published:** 2023-05-12

**Authors:** Pupalan Iyngkaran, Fahad Hanna, Sharon Andrew, John David Horowitz, Malcolm Battersby, Maximilian Pangratius De Courten

**Affiliations:** ^1^Heart Failure & Cardiac Imaging, Torrens University, Melbourne, VIC, Australia; ^2^Werribee Mercy Sub School, School of Medicine, University of Notre Dame, Werribee, VIC, Australia; ^3^Public Health, Torrens University Australia, Melbourne, VIC, Australia; ^4^Institute of Health and Sport, Victoria University, Melbourne, VIC, Australia; ^5^Basil Hetzel Institute for Translational Health Research, University of Adelaide, Adelaide, SA, Australia; ^6^Flinders Health and Medical Research Institute, Southern Adelaide Local Health Network Mental Health Service, College of Medicine and Public Health, Flinders University, Bedford Park, SA, Australia; ^7^Mitchell Institute, Victoria University, Melbourne, VIC, Australia

**Keywords:** congestive heart failure, Flinders program, mixed methods research methodology, protocol, risk assessment, self-management

## Abstract

**Introduction:**

Congestive heart failure (CHF) causes significant morbidity and mortality. It is an epidemic, and costs are escalating. CHF is a chronic disease whose trajectory includes stable phases, periods of decompensation, and finally palliation. Health services and medical therapies must match the various patient needs. Chronic disease self-management (CDSM) programmes that are patient-focused, identify problems and set actionable goals that appear as a logical, cost-friendly method to navigate patient journeys. There have been challenges in standardising and implementing CHF programmes.

**Methods and analysis:**

*SELFMAN-HF* is a prospective, observational study to evaluate the feasibility and validity of the *SCRinHF* tool, a one-page self-management and readmission risk prediction tool for CHF, with an established, comprehensive CDSM tool. Eligible patients will have CHF with left ventricular ejection fraction <40% and commenced sodium glucose co-transporter-2 inhibitors (SGLT2-i) within 6 months of recruitment. The primary endpoint is the 80% concordance in readmission risk predicted by the *SCRinHF* tool. The study will recruit >40 patients and is expected to last 18 months.

**Ethics and dissemination:**

This study has been approved by the St Vincent’s ethics committee (approval no. LRR 177/21). All participants will complete a written informed consent prior to enrolment in the study. The study results will be disseminated widely *via* local and international health conferences and peer-reviewed publications.

## 1. Background

Congestive heart failure (CHF) causes significant morbidity and mortality. At least 50% of patients have one or more comorbid conditions such as type II diabetes, coronary heart disease, chronic kidney disease, hypertension, and others. Patients diagnosed with CHF take multiple medications and prescribed complex treatment regimens. Good health literacy is needed to action diet, complex self-management skills, and maintain compliance life-long (1, 2). In addition, interacting effectively with health systems is required for lifestyle maintenance, behavioural changes, and consolidation of chronic disease self-management (CDSM). Patients and health system factors can act as barriers to optimal care, including developing new comorbidities, geography (isolation and remoteness), language, culture, education and socio-economic disadvantage, social supports, and negative attitudes towards establishments (3). Hospital readmissions are high, with 25–50% occurring within 1–6 months of discharge; this largest cost contributor is also preventable and amenable, as studies have demonstrated the care process (4). Better healthcare delivery systems could be achieved by the addition of patient self-management capabilities within programmes that meet patient needs and link health services (4, 5).

Chronic disease management and CHF programmes share similar care domains, one aspect is CDSM. Despite setbacks, there is optimism that CDSM will provide increasing importance to CHF programmes. Nevertheless, to an increasing extent, translational issues such as the containment of resource utilisation and cost blowouts must be factored into the aims of CDSM. These are, however, not the primary aims of large randomised controlled trials. These translational issues are better understood when exploring a patient’s journey. When patients seek health services, the acuity determines if interactions are in the community or *via* acute services such as ambulance, emergency, and hospital admissions. Following acute care, patients then transition back to the community. This process creates new information, new members of a health team, and may also create silos. There are CHF patients that inadvertently utilise the acute pathways and are potentially preventable admissions. It is thus vital that CDSM research aligns with clinical and administrative realities.

To acquire and action CDSM, most generic frameworks have three tiers: (a) four goals: performance mastery, modelling, interpretation of symptoms, and social persuasion; (b) three tasks: medical management, role management, and emotional management; and (c) five skills: problem solving, decision-making, resource utilisation, forming a patient–healthcare provider partnership, and taking action (3, 6–11). Models, processes, and systems of care are pathways to delivering disease management programmes (5). In theory, CDSM should assist these programmes; however, structural deficiencies exist for CHF. Nonetheless, tremendous gains have been documented with CDSM (1, 2, 12), disease management (13–17), and performance improvement strategies (1, 2, 4).

In relation to gaps that need attention, heterogenous patient populations, clinical scenarios and “one shoe fit all” models are important examples (11). In this context, programmes that balance comprehensiveness by identifying unique client problems set goals to complement guideline-based care with resources (7–9, 11, 13), link to broader health channels, and finally, contribute to risk stratification and readmission reduction will contribute to CDSM usefulness. On the latter, triaging CHF can be time-consuming and resource-intensive (10). CDSM programmes should also target this as an important performance measure. The *Self-management in Heart Failure Study (SELFMAN-HF) and Screening in Heart Failure risk stratification tool (SCRinHF)* explore these issues and are described in more detail.

## 2. Methods

### 2.1. Aim and scientific hypotheses

**Figure 1:**
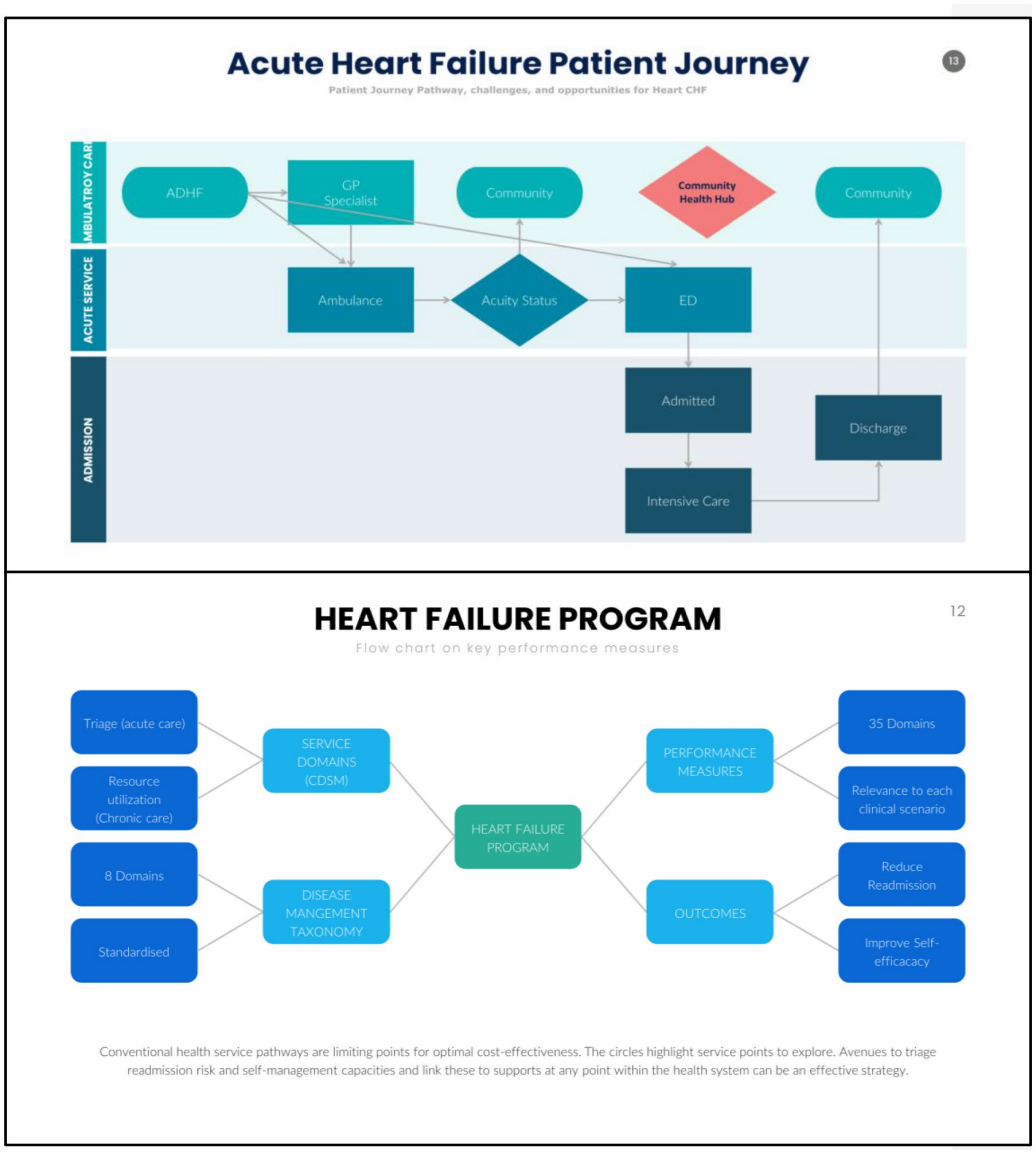
Patient journey pathway, challenges, and opportunities for heart failure.

**Figure 2:**
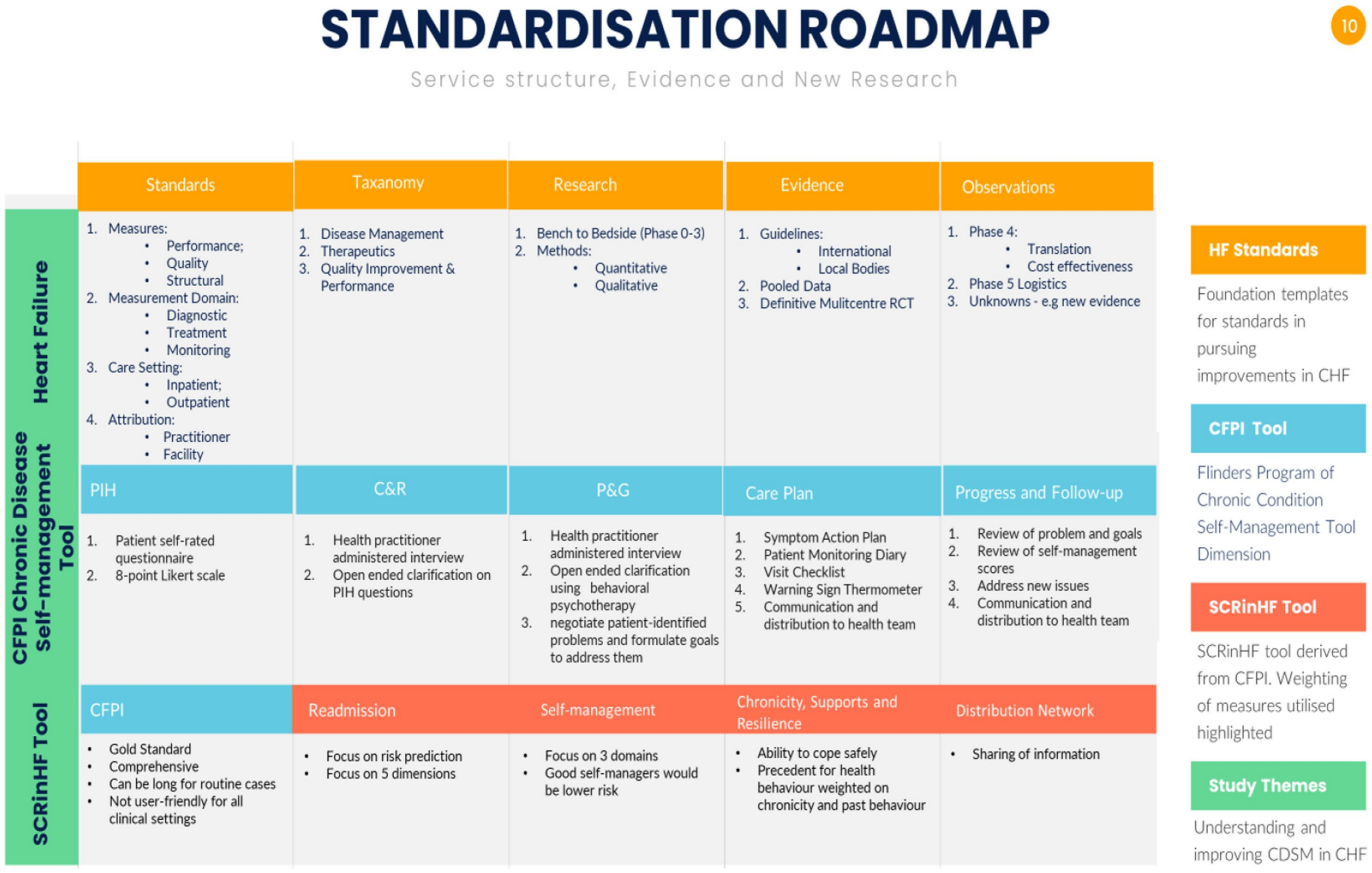
Summary of standardisation structures for evidence and clinical services. **(A)** Heart failure structures for clinical standards, taxonomy domains to build systems, research methodologies to gain evidence that eventually lead to guidelines. The subsequent steps are observations on: Phase 4 translation to trial type populations, cost-effectiveness to the general population; Phase 5 is a newly coined term to explore logistics in service delivery; the final point are unknowns such as vulnerable populations, new findings that require a bench to bedside approach of gathering evidence and testing these hypotheses in a Phase 3 randomised trial. Population level. RCT – randomised controlled trial [adapted from references (1, 2, 12–16)]. **(B)** Chronic disease self-management is standardised around the Flinders Program of Chronic Disease Condition Management (CFPI). Partners in Health (PIH) are 12 self-rated questionnaire patient complete to assess self-management knowledge, attitudes, behaviours, and impacts of their chronic condition; Cue and Response Interview (C&R) is administered by health workers using an open-ended dialogue on the same questions as the PIH, and rated from the health workers perspective, and shared with the patient; Problem and Goals assessment (P&G) is health worker tool utilising behavioural psychotherapy, open-ended questions negotiate patient-identified problems and formulate goals to address them; care plan records and scores behavioural changes, monitors and progressively implements at patients pace. Barriers, strengths, and priorities identified through PIH, C&R, and P&G are incorporated into fully negotiated care plans [adapted from reference (11)]; the SCRinHF tool extracts and focuses on five domains from the CFPI. As it is a screening tool, domains tested can be re-explored should a patient be identified as at high risk and require more intensive support.

The *Self-management in Heart Failure Study* assesses the feasibility and validity of a short form *SCRinHF* tool (6), with the established comprehensive *Flinders Program of Chronic Disease Self-management* (Flinders Program or CFPI) (18–21) for participants who were prescribed an SGLT-2 Inhibitor for CHF. In this, we hypothesise: first, guideline level of care as established by Ref. (1, 2, 12–17), and CDSM can be used to enhance the delivery and uptake of guideline-based care (22–26); second, to achieve this, we have to define clinical pathways. In this study, the model of care is based on patients utilising community cardiologists with community-based triage (Figure 1); third, a short form CDSM tool (*SCRinHF*) will improve efficiency in delivering CDSM programmes without compromising clinical outcomes as compared to the CFPI (Figure 2). The *SELFMAN-HF* study thus aims:

- To examine the relationship between CDSM and CHF-related health outcomes over a 12-month period. The CDSM tools that will be used are the CFPI and SCRinHF (Table 1). The study will establish the feasibility of use and the concurrent and predictive validity of the SCRinHF when compared to the CFPI in predicting self-management, clinical measures, and predictive measures of health outcomes (hospital readmissions) in CHF and their limitations and strength

**Table 1:** SCRinHF heart failure readmission risk scoring tool.

Domains of care	Heart failure ambulatory readmission risk dimensions of care	#Yes	No	*D
1. Baseline readmission risk	Comorbid risk [Ref (1–3)]	1–3+	0	
	Managing ADL	1	0	
	Adequate Social supports	1	0	
	Compliance	1	0	
	Mood – neurovegetative, psychological [Ref (4)]	1	0	
2. Living at home (skills & goals) Self-care maintenance Self-care management Self-care confidence/efficacy	a. Do you know “how to (skill).to achieve (goal)”… [Ref (5, 6)]			
	Problem solve – e.g. (i) monitoring;	0 or −1	1	
	Decision making question	0 or −1	1	
	About physical function – e.g. (i) exercise	0 or −1	1	
	b. Do you know “what to do if (skill) to achieve (goal)”…			
	Resource utilisation, e.g., (i) monitoring with action	0 or −1	1	
	Form patient provider partnership, e.g., (i) engage health system	0 or −1	1	
	Action planning when self-tailoring	0 or −1	1	
	c. Do you know “how confident you are (skill)…when faced with (goal)”			
	Has client previously received Rehab/education? State level of Self-Care Confidence (SR, SE, TI, TE) – e.g. (i) adherence to diet (ii) compliance	0 or −1	1	
3. Supports for living at home	Do you need additional services [Ref (7–10)]	0 or −1	1	
4. Chronology	Presentation of CHF or comorbidity acute (1) or subacute-chronic (0). (If acute go to long-form; cood with inpt team).	1	0	
5. TOTAL SCORE (NB// minus score given if excellent self-care capacity or support)				
6. HF Team (HFT)	15. Correspondence to: (dn, gp, ot, n, p, ph, r, shf, so, others)			
Service delivery needs				
Score ≤ 1 in each dimension or requires < 1 domain of care	Score = 2 in at least 2 or more for any dimension or > 1 domain of care	Score ≥ 3 for any dimension or > 2 for a dimension or domain of care		
Patient has low re-admission risk and can self-manage. Reassess bi-annually	Patient has moderate readmission risk and may have limited self-care capability	Patient is likely to be a high risk of readmission and probably does not have capacity to self-care independently		
Short-term allied health support and self-care education may be appropriate. Patient may be a good candidate for technology-assisted out-patient HF programs	Medium to long-term allied health support and self-care education may be appropriate. Patient may be a candidate for technology-assisted out-patient HF programs	Long-term allied health support and nurse-led out-patient support are likely to be needed. Patient is unlikely to independently self-care.		
Tailored resources required				
Domain combinations (C; H; T)	Dimension hierarchy (R; S; A)	Duration: (S; M; L)	Notes: -Intervention Model (I) - Other consideration	
1. 2.				

SCRinHF tool previously published (6). Abbreviations and operating manual refer to Supplementary Appendix.

## 3. Study objectives

The *SELFMAN-HF tests the feasibility and validity of the SCRinHF tool,* comparing the novel one-page tool to the gold standard Flinders Program of Chronic Condition Self-management Tool (CFPI). Feasibility: there are no prior comparisons of short and long form CDSM tools applied to CHF. Validity: first, this will explore comparisons of the SCRinHF to the CFPI in a range of measures including, self-management, clinical progress, and predicting 12 months major cardiovascular outcomes including readmission; and second, the Delphi method to attain peer acceptability and expert consensus on the *SCRinHF* tool.

## 4. Patient cohort and selection criteria

A minimum of 40 patients with CHF who meet the inclusion criteria will be offered an opportunity to participate. In uncertain cases, subjects will be considered eligible if the review of medical records demonstrates that they have a CHF diagnosis based on ACC/AHA and National Health Data Dictionary standardised definitions (1, 2). The project physicians will perform this. An independent physician will review uncertain cases.

- Eligible patients will be: aged over 18 years; able and willing to provide informed consent; started SGLT-2i within 6 months - for systolic HF (echocardiographic EF <40%); receive one aspect of CHF care within a defined study health corridor.
- Patients will be excluded: If concerns are raised by any medical staff, the patient has a life expectancy of ≤6 months whilst receiving palliative or nursing home care. Cognitive status and dementia will not be a contraindication if there is consent from a caring relative or legal guardian and the ability to complete the CFPI; have a significant neurological/cognitive impairment or are unable for any reason to provide written informed consent; do not usually reside within the region (or for whom no follow-up data can be obtained); started SGLT2-i for more the >6 months.
- *Health Staff* will be sent surveys if they manage chronic diseases, including CHF and are willing to complete an Assessment of Chronic Illness Care (ACIC) questionnaire.

## 5. Study design

### 5.1. Study objectives, measures, and endpoints

The *SELFMAN-HF* is a prospective, observational case-cohort study, in three phases: *Phase 1,* is defining the geographical boundaries of the community cardiology clinic in Western Melbourne, Australia, *and the* development of appropriate clinical indicators, study design, and registry; *Phase 2,* is the assessment of data and recommendations; and Phase 3, the Delphi methodology process. The measures and endpoints are highlighted for each sub-section.

### 5.2. Study mapping, development of appropriate clinical indicators, study design, and registry (Phase 1)

The geographical area was defined *via* an internet search, supplemented by direct communication with local council health agencies and Victorian state tertiary hospital jurisdictions. We reviewed publications within our group, the heart foundation, CSANZ and European and American Cardiac societies for self-management tools, readmission risk factors, and chronic disease assessment tools. As these areas have been greatly studied, we felt it reasonable to use consensus works as the foundation principles to consolidate and build from. The Krumholtz taxonomy of chronic disease management (12), Flinders Program of chronic disease self-management or CFPI (6–8), and established chronic condition assessment tools* will be used to standardise data collection (1, 2, 5, 11–16) and recording through the service channels [*previously published in Tables 1–3 in Iyngkaran et al. (3)].

### 5.3. Assessment of study data, measures, recommendations, and intervention (Phase 2)

**Figure 3:**
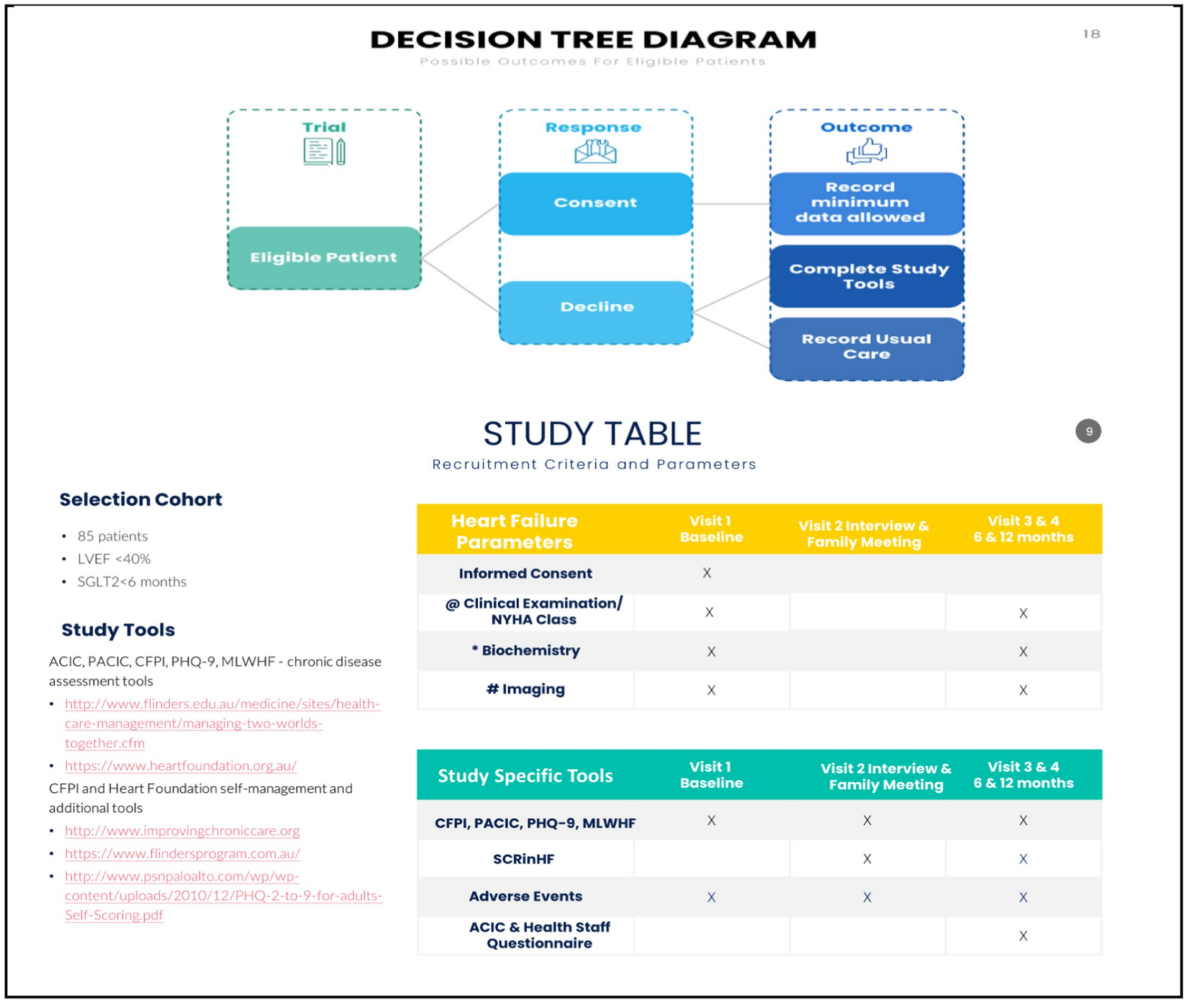
Study parameters and flow diagram. @ clinical consult, NYHA, 6MWT. # Echocardiography, ECG, CXR, ESE. *(FBE, EUC, lipids, HbA1c, Ca Mg, Alb, acute phase); UACR, NT-ProBNP. 6MWT, 6-min walk test; BP, blood pressure; eCDSMP, electronic chronic disease self-management programme; CHF, congestive heart failure; COPD, chronic obstructive pulmonary disease; CR, cardiac rehabilitation; CKD, chronic renal impairment; CXR, chest x-ray; DM, diabetes mellitus; EUC, electrolytes and kidney function; ESE, exercise stress echocardiography; ECG, electrocardiography; FBE, full blood examination; HbA1c, glycosylated haemoglobin; HFpEF, heart failure preserved ejection fraction; HFrEF, heart failure reduced ejection fraction; LVEF, left ventricular ejection fraction; MTWT, “Meeting Two Worlds Together”; MLHF, Minnesota living with heart failure; NT-pro-BNP, brain natriuretic peptide; NYHA, New York Heart Association; PACIC, patient assessment of chronic disease capacity; RCT, randomised controlled trial; RHC, right heart catheterisation; TBA, to be advised; UACR, urine albumin creatinine ration; yo, year old.

Details of the CFPI, heart foundation cardiovascular chronic disease tools, and patient assessment and chronic illness care satisfaction questionnaire (PACIC) are previously summarised [Table 3 in Iyngkaran et al. (3)] and published (3, 7, 8, 11). The CFPI tool and PACIC will be used to obtain information on patients’ CDSM capacity and patient satisfaction informing the CHF journey over 12 months. The PACIC is in three parts, where self-management assumes a component of the third category. Other study measures include the assessment of participants’ clinical changes in CHF and general wellbeing through the 6-month CFPI score, NYHA class, MLWF and 6MWT, and plasma NT-proBNP (Figure 3). Data on readmissions and mortality will be obtained from patient interviews and, if possible, healthcare records from main health providers, hospitals, primary healthcare (PHC) records, and community cardiac services. Cost–benefit analysis will be determined using standard billing criteria for public hospital funding Medicare Australia. These data will be compared with any ongoing prospective works on the CHF patient journey in Victoria. All enrolled patients will receive routine medical care as per Heart Foundation guidelines 2018 (27, 28). No additional investigations will be required from usual care. Comparing the long form CFPI and short form (*SCRinHF* tool) – trained staff who administer the CFPI will record the time to complete the process. The *SCRinHF* tool (Table 1) will be completed by an independent staff from those who filled in the CFPI. They are blinded to patients and will receive access to raw data on patients. The time to complete this will also be recorded.

### 5.4. Delphi methodology (Phase 3)

We aimed to conduct a Delphi Survey through a workshop, including a SCRinHF Trial Focus Group. A series of questionnaires will allow experts to develop ideas on the SCRinHF tool and potential future developments. The process will choose a facilitator, identify experts, define the problem, go through three rounds of questions, and finally act on the findings. The specific question will be formulated after the results of the cohort study are synthesised.

### 5.5. Study population/ recruitment of subjects / setting

The study setting of Western Melbourne defined the catchment although recruitment will only take place at community cardiology outpatients and support services for CHF. Recruitment will occur over a 6-month period. Patients identified will be referred to the study case manager for consent, and thetime will then be arranged to review the CFPI, PHQ-9, MLHF, and PACIC. Baseline demographics, tests, and questionnaire scores (Figure 3) will be recorded. All staff will be healthcare workers within community cardiology and aligned services. Participants will be recruited over a 12-month period, from September 2021 to September 2022, and followed for 12 months, ending September 2023. Health staff from primary and tertiary care managing CHF patients will be approached to complete the ACIC, between September 2021 and September 2022.

### 5.6. Participant assessments

- Baseline self-reported demographics and past medical history

A self-report questionnaire supplemented by a review of the patient’s medical records will inform baseline parameters collected: (1) determining clinical stability, (2) identifying key clinical, medication-related, and social issues, (3) assessing the status of key cardiovascular and CVD-related risk factors, and (4) recent hospitalisations (NB// Medical information from cardiology interaction will be extracted from recent clinical review).

- *Demography:* age, sex, marital status, social support, socioeconomic status (income), primary residence, mode of travel, and language and cultural group the patient identifies with
- *Behavioural factors:* smoking status, alcohol consumption, dietary behaviours, amount of physical activity, and sedentary time in the previous week
- *Health:* primary healthcare arrangements, co-morbidities, medications, primary care physician, pharmacy, assess to prior medical events, medical conditions/diagnoses, current treatments/medication regimes, prior hospitalisations, and procedures, with a particular focus on cardiovascular events, renal disease and diabetes, and self-reported history of hypertension, dyslipidaemia, and family history of coronary heart disease (CHD).
- *Clinical Assessments: will include* current symptom profile, New York Heart Association classification, height, weight, waist and hip circumference, blood pressure, cardiovascular and respiratory system assessment, and 12-lead electrocardiogram (ECG).
- *Psychosocial Status:* Psychosocial status will be assessed utilising standardised questionnaires, focused specifically on depression as assessed by the adapted PHQ-9 depression inventory and CFPI tool.

- Biochemical assessments

Pathology tests will be undertaken by accredited laboratories with standardised reference ranges; evaluation will be on current guidelines. All tests will be recorded directly from laboratory reports to standard CRFs. Investigations undertaken at baseline, 6 and 12-month follow-up include: fasting lipid profile (total cholesterol, high-and low-density lipids, and triglycerides – mmol/L); glycosylated haemoglobin (HbA1c), urinary albumin: creatinine ratio (mmol/L), B-type natriuretic peptide (NT-proBNP), high-sensitive C-reactive protein (hs-CRP), and renal and liver function tests (serum creatinine, urea, sodium, potassium and estimated glomerular filtration rate, bilirubin, ALT, and GGT).

### 5.7. Data collection and storage

Data will be collected on a standardised case note extraction form (CRF) by trained staff. Information will be accessed by self-report questionnaire and multiple sources including hospital records, primary healthcare clinic records, specialist databases, and record systems maintained by visiting district medical officers. The period of interest for data collection will be 0–12 months following the initial commencement of SGLT2-i. Data definitions will be standardised and widely accepted case and outcome definitions as outlined in the ACC Clinical Data Standards will be used (29). A locally convened panel of the research team will review cases that demonstrate ambiguity in data definitions or outcome data, and consensus sought. Only when two investigators agree will the data be recorded. All data will be de-identified when transferred for analysis, and subsequently will be stored within locked files at the Institute/University research office.

### 5.8. Participant follow-up

Clients will be followed up to determine subsequent hospitalisation, major medical events, and interventions at 6 and 12 months. Similar ICD codes for acute CHF will be used for screening information. Data extraction will include a combination of case notes review, medical databases, and contact with PHC and clients directly. Participants requesting changes in answers provided will be discussed at this or any interval to raise this point.

### 5.9. End points

The study endpoints for each phase are: Phase 1 – documentation of health journeys of patients; Phase 2 – interrogation of CFPI and SCRinHF tools for self-management capabilities and readmission risks; and Phase 3 – all data will be accumulated and discussed in the Delphi process. The primary endpoint is the readmission risk as scored by CFPI and SCRinHF and the correlation between the two tools. In addition, the following data will be collected: detailed maps of individual patient journeys and a patient and staff questionnaire on the process. Validation and times to complete the short and long forms will be recorded. The primary endpoint is 80% concordance in MACE and medical events between CFPI and SCRinHF tools. Secondary endpoints are data for cost analysis, and to inform the focus group in planning an RCT for efficacy, validity, reliability, and variability.

### 5.10. Data collection, management, CFPI training, and standardisation

Trained study investigators will collect study data, and completed CRFs will be checked for accuracy when entering information into a computer web-based interactive password-protected database administered by Mitchell Institute. On signing consent, participants will be given a unique study identifier, and information collected will be de-identified at source and a study identification used. Programme coordinators will conduct routine quality control. All documentation and case report forms associated with the study will be kept for a minimum of 15 years from the completion of the study. In accordance with the ICH GCP notes for Guidance on Good Clinical Practice, it is anticipated that the data will be stored indefinitely according to the Standard Operating Protocols.

*Training and standardisation:* Staff conducting the study will receive training and relevant documentation and ongoing professional support from the relevant universities (Victoria, Flinders, Notre Dame Universities). Staff delivering the ACIC, PACIC, Heart Foundation, and associated tools will undergo an accreditation process and be assessed as competent against the current standard. In addition, this project requires access to data housed and maintained in hospitals and PHC. Hospital separation data, hospital, and PHC records will be sought. If records are housed within independent services, appropriate consultation will be undertaken with the services themselves. Formalised consent processes as directed by those services will be followed. The potential for variability in data recording is noted and will be standardised by training staff on the ICD-10 classification for CHF and ACC/AHA guidelines for key performance indicators (5, 13–16, 29). Areas of ambiguity will be discussed with at least two members of the steering committee and recorded if there is agreement from both parties.

### 5.11. Adverse events

We do not anticipate any adverse events from this study. All procedures are in place as per local institutions’ guidelines to address issues relating to client dissatisfaction or concerns. Any medical complication will be addressed by the treating physician/institution as part of standard care.

### 5.12. Expected outcomes

The primary outcomes are to achieve concordance in readmission risk between CFPI and SCRinHF, and the number of patient readmissions to the hospital. The secondary outcome is to achieve convergent validity between CFPI and SCRinHF and potential economic cost benefits using the SCRinHF tool.

### 5.13. Randomisation/blinding

Not applicable for Phases 1–3. Trained study staff completing the SCRinHF tool will have access to raw patient data, but be blinded to the patient when completing the tool. Following Phase 3, a focus group will convene to finalise the SCRinHF trial and study tool. Randomisation strategies will be a priority for any future studies.

### 5.14. Study timelines and follow-up project milestone

Study recruitment will commence in September 2021 and end in September 2022 (12 months). A census of all outcome data will be conducted in December 2022. There should be >40 potentially eligible patients over the 12-month recruitment and follow-up period.

### 5.15. Statistical aspects and data analysis

The study cohort assesses feasibility, which will allow appropriate parameters to determine sample size power calculations for future studies that will utilise a controlled design. Initial data analysis will investigate the distribution characteristics of each primary and secondary outcome measure and determine either a parametric or semi-parametric statistical approach to main data analysis. A combination of descriptive statistics, linear mixed models, and generalised estimating equations will be used to calculate and present data. If continuous outcome data are approximately normally distributed, then linear mixed models (LMMs) will be used to examine change over time for each measure at intra-and inter-individual levels. If the distribution of outcome data is more amenable to a semi-parametric approach, then we will use generalised estimating equations (GEEs) to calculate an average estimate of the intervention effect for the cohort. Descriptive statistics for baseline demographics and clinical characteristics will be presented as means (standard deviation) for continuous data and count (percent) for categorical data. As an example, the previous publication provides estimates for pilot sample size calculations for categorical data. A future RCT of 200 patients in each arm for effects size of 25% reduction in readmission at 12 months established an event rate of 1,600 readmissions at 12 months (25–50% historical rate at 1 and 6 months), 90% power, and 5% two-sided significance, and 25–75 patients are published recommendations for small to large, standardised effect sizes (30). With the introduction of SGLT-2, the population level effect size is not known, and we have chosen >40 patients with an LVEF <40% and a NYHA class of 2 or 3. The event rates are well documented as 25% for 1-month readmission and 1-year mortality. Follow-up at 12 months predicts >100 admissions and > 10 mortalities. Interim analysis will ensure that this modelling supports the study goals.

### 5.16. Patient and public involvement

The development of this study was informed by the high rate of heart failure readmissions and poor uptake of CDSM programmes. No patients were involved with the study design; however, both experience and the published literature supported these directions. Patients will be provided feedback upon study completion. Public involvement has occurred during the formulation of the study and generic tools such as the CFPI. Further efforts will be made to provide community engagement, especially *via* focus groups.

### 5.17. Ethics and dissemination

This study has been approved by St Vincent’s ethics committee (approval no. LRR 177/21). All participants will complete a written informed consent prior to enrolment in the study. The study results will be disseminated widely *via* local and international health conferences and peer-reviewed publications.

## 6. Discussion

Congestive heart failure programmes utilising CDSM raise broad questions about the paradigms for evidence, followed by clinical translations for health services research. In contrast, pharmacological and device-based therapies have achieved class 1 and Level A evidence; similarly, physical exercise and organised programmes including cardiac rehabilitation have secured evidence in clinical guidelines (1, 2, 5, 12, 27, 28). CDSM is a complex intervention that aims to achieve better contextual self-management efficacy for an individual’s chronic ailment. CHF clinical trials using therapeutics predominately explored *major adverse cardiovascular events* (MACEs) as primary endpoints, and with clinical pathophysiological changes, cost, quality, and disability of life, as secondary endpoints. Diagnostic tools and instruments to test CHF and life measures are also established (13–17, 29). The established evidence base is consolidated into organised frameworks such as guidelines (1, 2, 12), taxonomies (5), and processes of care (12–17) (Figure 1). The study approach we undertake for CDSM in HF utilises a mixed-methods approach, whereas, with qualitative research, it is harder to achieve higher levels of evidence. In this study, the first phase is an evidence review to standardise the trial protocol on current published guidelines; the second phase balances the qualitative and quantitative arms to reflect the true nature of the processes in question. For example, in the 2020 guideline revision of CHF clinical measures, patient self-care education was changed from a performance measure to a quality measure, citing concerns about the limitations in the evidence for improved outcomes (22–25). It is thus time to ponder on how to best rebuild the evidence. In addressing the specific challenges for this study and also in creating hypotheses for future studies, we highlight the following strengths and limitations:

A strength of this study is the ability of the observational sample size to inform a future randomised study, by the event rates anticipated with the CHF inclusion criteria (LVEF <40%). In Australia, an SGLT-2i can be provided to all patients with NYHA class II and LVEF <40%; thus, its use requires a standardised baseline, and it requires education on self-management to use it safely. In addition, we were also able to leverage on previous works which established performance measures (5, 12, 13, 15, 16, 29) some updated (14), to inform and extract parameters for this study.

An important limitation in study-specific tools is questionnaires that have qualitative and quantitative components [Tables 2, 3 previously published (3)]. The frameworks for CDSM and CPFI (11) have been previously detailed. These goals (11, 19–21) and established CHF readmission risk scores were foundations on which the SCRinHF tool was modelled. The limitations of traditional tools, including patient fatigue (45–60 min to complete), were the impetus for the development of a one-page tool; overlaps in questions between tools and investigators were the motivation to train end users to populate other tools with overlapping questions where appropriate [previously published Table 2 Iyngkaran et al. (3)]. Monitoring clinical changes with CHF is also important. The balance between quick and less accurate assessments such as NYHA classification, general chronic disease scores, and CHF-specific scores can impact clinical standardisation and require thoughtful considerations (30–34). These points will also be factored in when informing a larger definitive RCT.

*Generic limitations and strengths*: The study design is hypothesis generating, and results may reflect a degree of subjectivity. Generalisability of results will not be answered in this study. Standardising training of study case workers that arise from different allied health backgrounds, that bring a different perspective to health provision will be difficult. There was no background data to draw from in determining sample size and ensuring the validity and reliability of the qualitative tools including PACIC and PIH scales. The level of engagement with the wider communities has also been limited at this stage. This will however be addressed when there are clear working hypotheses, with an improved understanding in this area, that could contribute to the design of a larger more focused study, involving community participation. The Delphi method will be published when the data are available from Phase 2.

In conclusion, the specific challenges highlighted will be addressed in the creation of hypotheses for future studies. Areas that will be challenging include the CDSM programme for clients from diverse/lower educational and socioeconomic backgrounds where the programme may have variable effectiveness. Delivery to clients placed at the apex of the health pyramid and engagement in primary care has shown the greatest health impact (8, 9, 21). With scoring, patient perceptions of illness are qualitative and hard to standardise. Scales such as The European Heart Failure Self-care Behaviour Scale (EHFScBS) and Self-care Heart Failure Index (SCHFI), amongst 14 other HF self-care instruments, have proven reliability and validity; however, they do not adequately address co-morbidities (8, 9, 21). Demographic heterogeneity is also greater outside Phase 3 trials. The themes that emerge at the population level include cultural sensitivity, patient-centred care, creating specific policies for disparity, emphasis on accuracy and detail of information, engaging extended families, and providing tools to facilitate health provider–patient relationships within the larger socio-cultural system (5). All these factors will be relevant as this study evolves.

## Strengths and limitations

- Explores a simple user-friendly self-management and readmission tool.
- Self-management programmes can be complex with few gold standard publications.
- Enrols a diverse cohort of congestive heart failure patients.
- Elements are hypothesis-generating, e.g., a scoring system for the *SCRinHF* tool has not been tested.
- Improves our understanding of self-management scores in heart failure but will require a larger study to corroborate findings.

## Author contributions

PI, FH, SA, and MD participated in research design, data analysis, and writing of the manuscript. PI, participated in performance of the research and data collection. PI, FH, SA, JH, MB, and MD provided advice and support. All authors have read and approved the manuscript.

## Funding

MB was co-inventor of the “Flinders Model of Chronic Condition Self-Management” and has received competitive and Federal Government funding for research in chronic condition self-management. All other authors have received government and non-governmental funding. None pose a conflict of interest for this publication.

## Conflict of interest

The authors declare that the research was conducted in the absence of any commercial or financial relationships that could be construed as a potential conflict of interest.

## Publisher’s note

All claims expressed in this article are solely those of the authors and do not necessarily represent those of their affiliated organizations, or those of the publisher, the editors and the reviewers. Any product that may be evaluated in this article, or claim that may be made by its manufacturer, is not guaranteed or endorsed by the publisher.
