# Supplementary material for: Comparison of short and long forms of the Flinders program of chronic disease SELF-management for participants starting SGLT-2 inhibitors for congestive heart failure (SELFMAN-HF): protocol for a prospective, observational study
Source: Front Med (Lausanne). 2023 May 12;10:1059735. doi: 10.3389/fmed.2023.1059735 (PMC10255353; doi:10.3389/fmed.2023.1059735)
Supplement: Supplementary file 1 [file Image_1.pdf]

Supplementary image 1 for Table 1

Figure 1b : We propose a framework for a clinical tool to assess congestive heart failure (CHF) readmission risk for ambulatory patients, by defining the actionable care areas to distribute resources following first and early encounters. The need for such a tool is evident following outpatients and new emergency department presentations. From experience among the successfully translated clinical tools in cardiovascular medicines has been CHADVASC-2 and HASBLED scoring system for atrial fibrillation (AF) which also shapes clinical management. A translatable tool for CHF has been less forthcoming. Fundamental differences in disease processes exist between CHF and AF however fundamental concepts in screening scores are firstly defining the risk, in this case readmission as opposed to stroke; secondly the domains and dimensions of care most influencing this risk; and finally the scoring system to execute the management strategy, with CHF a basket of options unlike fixed haematological strategies with AF.

A CHF scoring system has thus more interactive components, but the ease of use must none the less remain similar as with the AF scores. We have thus isolated the clinical assessment needs to 1) specific high risk readmission criteria: including, comorbidities, and efficacy 3) additional support domains required 4). 4) Final Score and health services support to be mobilised. We cite references for previously published work on each of the areas, individually [1-10]. A combined approach is the current focus.

ABBREVIATIONS: (Infrastructure – use large caps; Personnel – use lower caps)

Self-Care SKILLS:1) Problem solving 2) Decision making 3) Resource utilization 4) Form patient provider partnership 5) Action planning with Self-tailoring

Self-Care GOALS 1) Monitoring (G,M) 2) Monitoring with action (G) 3) Exercise (G,M) 4) RF Modification and preventive behaviours (G) 5) Engaging health system (G,M) 6) Compliance (G,M,P) 7) Diet adherence (G,M,P)

***D =** domain of care to action deficit. A/C = Ambulatory or Community; H = hospital; T = technology assisted CHF care; I = intervention category (potential overlap); HFT – heart failure team.

- C1 – GP; C2 – cardiologist; C3 – community allied health.
- H1 – ED; H2 – specialist clinics; H3 – hospital allied health.
- T1 – phone; T2 - mobile; T3 – internet.
- I1 – Case management; I2 – Chronic care model; I3 – discharge management; I4 – Multidisciplinary team; I5 - Complex intervention; I6 – primary or secondary care follow-up; I7- self-care.
- HFT - h = hospital; p = private; dn – district nurse; gp - general practitioners; n - nurse; ot – occupational therapist; p - physiotherapy; ph – pharmacy; ps – psychologist; r – rehab; shf = HF specialist; so – other specialist;

#Yes = dimensions of care to action within each health care domain. R = highest readmission risks; S = self-care; A = ambulatory care at home

- R1 – Comorbidity; R2 - Functioning (physical, occupational, perceptions on health (potential overlap), personal belief (potential overlap), psychological and social functioning (potential overlap); R3 – social supports; R4 – mood (neurovegetative, psychological); R5 – compliance.
- S1 - Patient Activation (items 1-3); S2 Delivery System Design/Decision Support (items 4-6); S3 - Goal Setting (items 7-11); S4 - Problem-solving/Contextual Counselling (items 12-15); S5 - Follow-up/Coordination (items 16-20). (SR – symptom recognition; SE – symptom evaluation; TI – treatment implementation; TE – treatment evaluation)
- A1 – independent; A2 – some supports; A3 – dependant.**Duration: S** - Short; M - Medium; L - Long-term.
